# Syncretic Grad-CAM Integrated ViT-CNN Hybrids with Inherent Explainability for Early Thyroid Cancer Diagnosis from Ultrasound

**DOI:** 10.3390/diagnostics16070999

**Published:** 2026-03-26

**Authors:** Ahmed Y. Alhafdhi, Gibrael Abosamra, Abdulrhman M. Alshareef

**Affiliations:** Department of Information Systems, Faculty of Computing and Information Technology, King Abdulaziz University, Jeddah 21589, Saudi Arabia; gabosamra@kau.edu.sa (G.A.); amralshareef@kau.edu.sa (A.M.A.)

**Keywords:** CNN, ViT-E, fusion features, Grad-CAM, XGBoost, ANN, thyroid cancer

## Abstract

**Background/Objectives:** Accurate detection of thyroid cancer using ultrasound remains a challenge, as malignant nodules can be microscopic and heterogeneous, easily confused with point clusters and borderline-featured tissues. Current studies in deep learning demonstrate good performance with convolutional neural networks (CNNs) and clustering; however, many approaches focus on local tissue and provide limited, non-quantitative interpretation, reducing clinical confidence. This study proposes an integrated framework combining enhanced convolutional feature encoders (DenseNet169 and VGG19) with an enhanced vision transformer (ViT-E) to integrate local feature and global relational context during learning, rather than delayed integration. **Methods:** The proposed framework integrates enhanced convolutional feature encoders (DenseNet169 and VGG19) with an enhanced vision transformer (ViT-E), enabling simultaneous learning of local feature representations and global relational context. This design allows feature fusion during the learning stage instead of delayed integration, aiming to improve diagnostic performance and interpretability in thyroid ultrasound image analysis. **Results:** The best-performing model, ViT-E–DenseNet169, achieved 98.5% accuracy, 98.9% sensitivity, 99.15% specificity, and 97.35% AUC, surpassing the robust basic hybrid model (CNN–XGBoost/ANN) and existing systems. A second contribution is improved interpretability, moving from mere illustration to validation. Gradient-weighted class activation mapping (Grad-CAM) maps demonstrated distinct and clinically understandable concentration patterns across various thyroid cancers: precise intralesional concentration for high-confidence malignancies (PTC = 0.968), edge/interface concentration for capsule risk patterns (PTC = 0.957), and broader-field activation consistent with infiltration concerns (PTC = 0.984), while benign scans showed low and diffuse activation (PTC = 0.002). Spatial audits reinforced this behavior (IoU/PAP: 0.72/91%, 0.65/78%, 0.58/62%). **Conclusions:** The integrated ViT-E–DenseNet169 framework provides highly accurate thyroid cancer detection while offering clinically meaningful interpretability through Grad-CAM-based spatial validation, supporting improved confidence in AI-assisted ultrasound diagnosis.

## 1. Introduction

The thyroid gland, an endocrine gland, is located in the front of the neck below the larynx. It is one of the largest endocrine glands, typically weighing between 25 and 30 g. The gland secretes two hormones: triiodothyronine (T3) and thyroxine (T4) [1]. These hormones play crucial roles in regulating metabolism and growth, affecting almost all body tissues. The thyroid gland and its associated hormones affect a wide range of body systems, including the cardiovascular and nervous systems [2]. Common symptoms of thyroid dysfunction include anxiety, impaired cognitive function, menstrual irregularities, rapid heartbeat, muscle pain, weight gain, and elevated cholesterol levels [3]. Some thyroid disorders, including certain types of thyroid cancer, have genetic components. Exposure to ionizing radiation, particularly during childhood, increases the risk of thyroid cancer. Iodine deficiency or excess, as iodine is essential for thyroid hormone production, can lead to thyroid disorders. Iodine deficiency causes conditions such as goiter, whereas excessive iodine intake can lead to hyperthyroidism [4]. Several autoimmune diseases, such as Hashimoto’s thyroiditis, are associated with hypothyroidism due to insufficient hormone production by the thyroid gland [5]. Graves’ disease also causes hyperthyroidism due to the thyroid gland secreting excess hormones [6]. A thyroid cancer diagnosis requires clinical and imaging evaluations. Several methods exist for diagnosing this cancer, including a thorough neck examination by a specialist to detect any abnormalities, such as masses or nodules [7]. Ultrasound images provide detailed images of the thyroid gland and help evaluate thyroid nodules by determining their size, characteristics, and location [8]. Blood tests measure the concentrations of thyroid hormones (T3 and T4) and thyroid-stimulating hormone (TSH) to assess overall thyroid function [9]. CT and MRI are used to evaluate thyroid cancer and determine its spread to lymph nodes [10]. Manual diagnosis of thyroid cancer has several limitations [11]. In the early stages, the clinical symptoms are mild and similar to those of other conditions, making manual diagnosis difficult [12]. There is no consensus among specialists on how to identify suspicious thyroid nodules or differentiate between aggressive and non-aggressive nodules [13]. AI techniques have proven highly effective for processing manual diagnoses from CT data, particularly in the early stages [14]. CNN models analyze ultrasound images to identify features indicative of thyroid cancer. These networks analyze massive datasets at high speeds and recognize features that are difficult to detect with the naked eye. AI models assist in analyzing and classifying image data and provide a unified interpretation of ultrasound images [15].

This study addresses a long-standing diagnostic challenge in the early detection of thyroid cancer: the thin, gray line on ultrasound images, where human assessment is inadequate and conventional deep learning algorithms are often clinically inaccurate. Although traditional convolutional neural networks can extract local patterns, they are often used with perceptual segmentation. They are used to isolate tissues and margins without grasping the broader anatomical picture necessary for an accurate diagnosis. This creates a significant discrepancy between statistical and clinical validity. We aim to move beyond incremental model optimization and strive for architectural coherence. Our goal is to develop a comprehensive diagnostic system that fundamentally integrates the detail-focused, locally interpretable deep learning with a holistic, relational interpretation, both of which are essential for interpreting the entire ultrasound landscape and making all predictions highly accurate and virtually interpretable.

This paper discusses different methodologies, tools, and results of previous works with the aim of detecting cancerous thyroid nodules.

Sujini et al. [16] presented DL models: a six-layer CNN and a VGGNet-16. A 6-CNN model was developed for efficient end-to-end analysis, and ultrasound images of the thyroid containing malignant and benign cases were used. The combined CNN-VGGNet-16 technique achieved an accuracy of 0.97. Li et al. [17] introduced a transformer fusing the CNet method for automatically segmenting malignant thyroid nodules. The CNet comprises a CNN branch with a Large Kernel Module for precise shape feature extraction and an enhanced transformer branch with an Enhanced Transformer Module for remote-pixel connectivity in ultrasound images. A Multiscale Module was used to combine features from branches. Zhang et al. [18] presented multi-CNN trained for thyroid disease classification. This study also explored strategies to enhance the diagnostic accuracy of CNNs by combining feature maps of different scales. The multi-CNN outperformed the standard single-channel CNN. It achieved 0.91 accuracy, 0.94 precision, and 0.90 recall for thyroid disease classification. Namdeo et al. [19] presented a model for thyroid disorder diagnosis. First, image and data features were extracted using neighborhood-based PCA. Two classification processes follow: a CNN for image classification and an NN for disease classification, both using features as inputs. The combined results were used to enhance the diagnostic accuracy. Naglah et al. [20] developed a system to extract complex texture patterns using CNN. The system integrates multiple channels for all inputs, merging the collected scans into the DL and utilizing various adjustable diffusion gradient coefficient values. The system achieved accuracies of 0.87 and sensitivities of 0.69. Li et al. [21] presented a CNN-based system for thyroid nodule recognition. It employs an enhanced U-Net segmentation method to isolate the ROI, optimizes the ROIs using image processing, and classifies them as benign or malignant using a CNN-Fusion network. The results showed strong performance, with segmentation and classification values of 0.855 and 0.86, respectively. Zhao et al. [22] trained and tested five different CNNs on thyroid nodules images. The study found that the CNN models had significantly higher diagnostic performance (AUCs ranging from 0.901 to 0.947) for thyroid malignancies. The ensemble model, which combined three of the best-performing CNNs, achieved the highest AUC value, indicating its effectiveness in diagnosing thyroid nodules. El-Hossiny et al. [23] presented a CNN for TC classification. The CNN architecture achieved 94.69% accuracy in thyroid carcinoma classification. Aljameel et al. [24] designed an Explainable-ANN model to classify thyroid nodules and identify predictive factors for malignancy. The SMOTEENN sampling method was applied to address the class imbalance. The Explainable-ANN model achieved an accuracy of 0.82 and an AUC of 0.86. Wu et al. [25] used three CNNs for classifying thyroid ultrasound images. In independent testing, the best-performing DL algorithm achieved AUCs of 0.829 and 0.779, respectively. Zhang et al. [26] studied an InceptionResNetV2-based framework that was developed and evaluated. The framework includes three multichannel models. It outperformed the ML methods, achieving an accuracy rate of 0.971 and a recall of 0.90. Wang et al. [27] presented a CNN for TC image analyzed for clinicopathological factors. Independent risk factors for TC, such as nodule size and BRAF gene mutation, were identified. The CNN achieved an AUC of 0.78. Rho et al. [28] presented a study to evaluate a deep CNN for distinguishing between malignant and benign thyroid nodules. The CNN was trained on ultrasound images of larger nodules (≥10 mm) and tested on smaller nodules (<10 mm). The CNN outperformed radiologists, achieving 0.832 accuracy, 0.383 specificity, and 0.66 in AUC. Vasile et al. [29] developed an ensemble method combining two DL models: one, called 5-CNN, and the other based on a repurposed and optimized VGG-19 architecture, for classifying thyroid ultrasound images. The ensemble CNN-VGG method achieved results surpassing both 5-CNN and VGG-19, with 97.35% accuracy and 95.75% sensitivity.

These studies collectively disclose three interdependent gaps in existing studies. First, most current frameworks are based on convolutional architectures that capture local image features but fail to capture global spatial interactions among ultrasound images. Second, most successful systems are black-box classifiers that offer no interpretability or visual justification for their predictions. Third, a comparatively small range of models attempts to combine feature extraction, global context modeling, and explainable inference into a single, unified architecture.

This study addresses these gaps. The proposed architecture enables local feature learning and long-range contextual reasoning to coexist within a single architecture by combining convolutional processing with vision transformer-based global attention mechanisms. More importantly, an interpretation was provided using Grad-CAM as part of the prediction pipeline. Rather than creating a plain label, the system creates spatially consistent visual explanations to indicate the areas that affect the diagnostic decision. The outcome is not just a classifier but something that can help clinical reasoning another step away; it is more of a digital assistant than a muted statistical engine. These explanations are already included in the updated manuscript to clarify the research gaps underlying the proposed hybrid CNN-ViT architecture.

The key contributions of this study are as follows:

- Development and evaluation of two new concurrent hybrid architectural models, ViT-DenseNet169 and ViT-VGG19. These are not clusters but integrated systems in which the ViT encoder and an optimized convolutional neural network backbone are trained together in continuous dialogue, enabling the simultaneous extraction of local features and their global contextualization.
- Integration of interpretability into core model processes. The Grad-CAM visualization method is integrated into the inference path, making the model appear as a transparent diagnostic partner rather than a black-box classifier. This allows for a graphical review of the model’s spatial inference, whether it focuses on the nodule core, invasive margins, or diffuse normal tissue, directly linking its decisions to identifiable acoustic biomarkers and establishing the necessary clinical confidence.
- Development and evaluation of hybrid systems combining XGBoost-CNN and ANN-CNN for the diagnosis of thyroid cancer ultrasound images.
- Development and evaluation of hybrid systems integrating features from multiple CNN and classifying them using XGBoost and ANN were developed and evaluated to support the visual diagnosis of thyroid cancer.

The remainder of this paper is organized as follows. Section 2 describes the materials and methods used, including the dataset, image enhancement, and the architecture of the proposed hybrid systems (Section 2.5 Hybridization of ViT-E with DenseNet169 and VGG19; Section 2.6. Inference and imaging of the attention mechanism). In Section 3, the experimental results are presented and discussed, and the performance of the ViT-CNN hybrid systems is analyzed in the conclusion (Section 3.5). The combination of convolutional and diagnostic accuracy, along with a graphical study of their decision-making processes, is presented (Section 3.6). Grad-CAM was used as an interpretive lens. Section 4 provides a comprehensive discussion and comparison of the performances of all models. Finally, Section 5 concludes the study by summarizing the main findings and their implications for future research.

## 2. Materials and Methods

**Figure 1:**
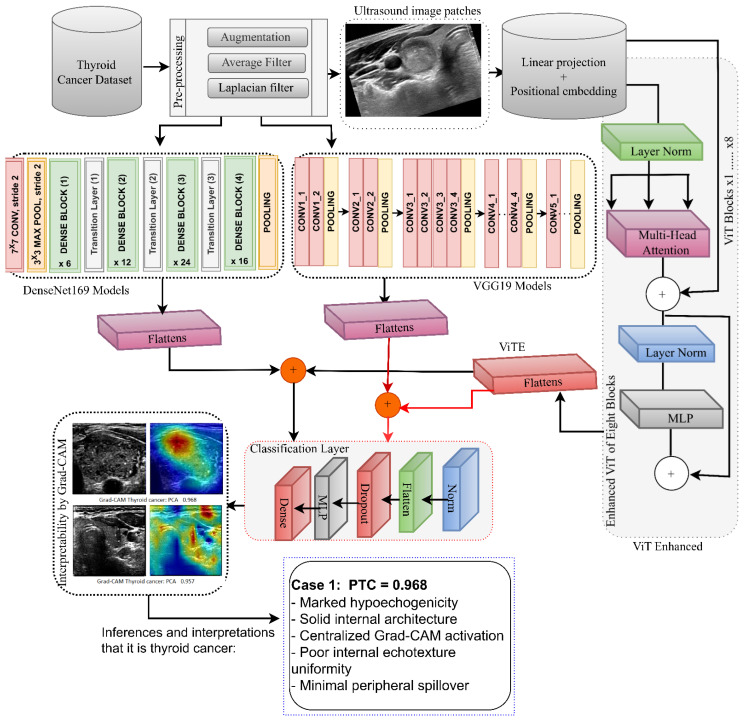
An interpretable end-to-end deep diagnostic architecture integrating dense convolutional encoding and vision transformer reasoning for robust thyroid cancer detection.

Figure 1 illustrates a sophisticated, comprehensive diagnostic system that combines convolutional neural network-based inference with transducers to detect thyroid cancer in ultrasound images. The process begins with specific preprocessing steps (enlargement, median filtering, and Laplace optimization) aimed at minimizing the impact of spotting noise while preserving the diagnostically relevant edges and internal structures. This is a crucial step because anatomically accurate input representation is essential for subsequent data interpretation.

Local morphological markers were simultaneously acquired using the DenseNet169 and VGG19 networks. These convolutional flows encode integrated fine-grained patterns, such as echo gradients, edge irregularities, and internal heterogeneity. Their outputs do not produce independent features; instead, they are integrated with the overall contextual representations learned by an optimized ViT network. Using linear projection and localized embedding, the ViT branch learns spatial relationship patterns across long distances within the gland, enabling a patch-level contextual interpretation beyond localized areas.

Interpretability is directly integrated via Grad-CAM, which operates on the last convolutional feature maps and redraws significance scores on the image. The resulting heat maps indicate the location and cause of the model’s presence, which may be focal lesion activation, peripheral capsule focus, or diffuse benign activity. Model attention and known ultrasound biomarkers, such as low-echoic nuclei or invasive edges, were spatially aligned to provide a checklist for clinical inference.

Overall, the figure demonstrates that interpretability is not a supporting visual display layer; rather, it is an inherent feature of the model structure. The framework combines the specificity of DenseNet169/VGG19 features, ViT-based global inference, and Grad-CAM annotations to enhance thyroid cancer diagnosis, with improved transparency and clinical reliability.

### 2.1. Description of Dataset

**Figure 2:**
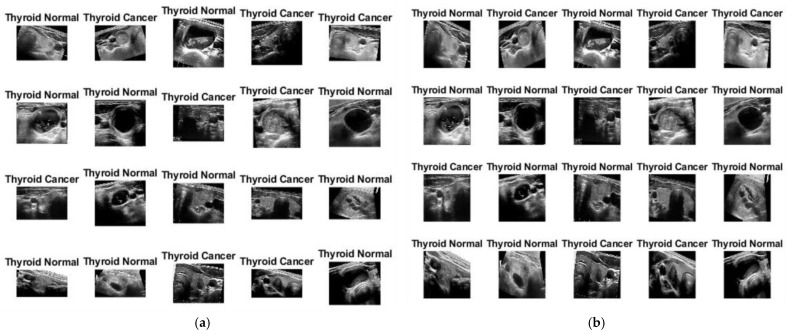
Sample thyroid images of the TC dataset. (**a**) Before enhancement; (**b**) after enhancement.

The thyroid cancer ultrasound dataset consisted of 7288 images [30]. This dataset was divided into two categories: TC (4006 images) and normal tissue (3282 images). This dataset was used to train and evaluate the proposed systems for diagnosing thyroid cancer. Figure 2a shows examples of ultrasound images from the thyroid cancer dataset.

This study aimed to detect and characterize the early stages of thyroid cancer by applying the XGBoost algorithm and an ANN based on CNN features to ultrasound images from the thyroid cancer dataset. The dataset consisted of 7288 images divided into two categories: thyroid cancer and normal tissue. The dataset was distributed as follows: 4006 images of thyroid cancer tumors and 3282 images of normal thyroid tissue. It should be noted that the dataset is unbalanced between the two categories; therefore, this issue will be addressed. To facilitate training and validation, the dataset was divided into two distinct subsets, as shown in Table 1, with 80% of the data allocated to the training and validation subsets in an 80:20 ratio, respectively. This partitioning strategy enables the algorithms to learn from most of the data while simultaneously validating its performance. Furthermore, a dedicated subset comprising 20% of the dataset was retained for the precise evaluation of the systems.

After retaining 20% of the 7288 images as a stable test set, we performed five-fold stratified cross-validation on the remaining 80% of the data to select the model and fine-tune the hyperparameters. Within each fold, four folds trained the model, while the remaining fold validated it, and the class ratios were maintained to preserve the TC/TN balance. Data augmentation (and any fitting steps, such as principal component analysis) was applied only within the training partition of each fold to prevent leakage.

### 2.2. Augment and Balance the Dataset

**Figure 3:**
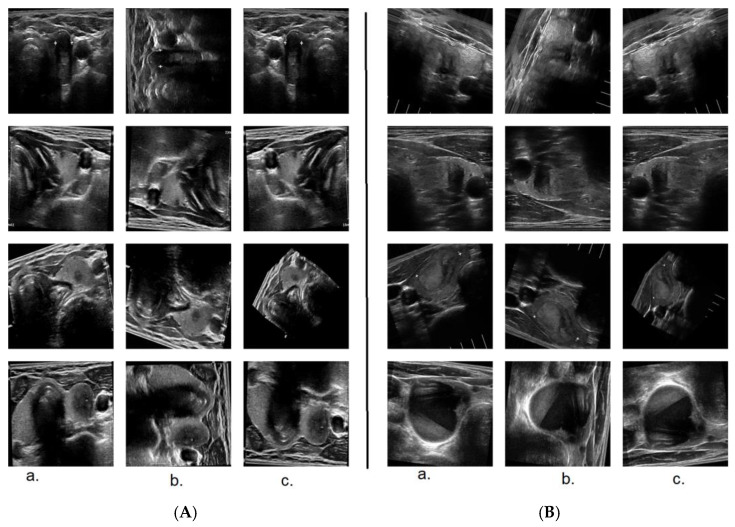
Representative ultrasound images of thyroid cancer and normal thyroid with corresponding data augmentation transformations. The first three columns display original ultrasound images of thyroid cancer. (**A**) Data augmentation technique: Number of images of a thyroid cancer class. (**a**), alongside augmented variants (**b**,**c**). The following three columns present original ultrasound images of normal thyroid tissue. (**B**) Data augmentation technique: Number of images of a normal thyroid class. (**a**), with their corresponding augmented images (**b**,**c**) generated through data augmentation techniques.

Data augmentation is a common method in image classification that increases the size of a dataset by creating new, slightly modified images from the existing dataset. This helps improve the model’s performance by providing diverse training samples. In the context of a TC dataset, data augmentation is particularly useful for two main purposes: increasing the number of ultrasound images and balancing the datasets [31]. One way to augment the data is by applying rotation, scaling, and flipping transformations to the original images. For example, the original ultrasound images were rotated by degrees (90°, 180°, etc.) as shown in Figure 3. This simulates the different angles at which the ultrasound images were captured. These transformations create new images similar to the original images but with different features [32]. The images were flipped horizontally and/or vertically. This mimics the orientation of the thyroid gland in different patients. Scaling involves resizing images to slightly different dimensions. This accounts for variations in the image resolution and aspect ratio. The translation shifts the images horizontally and/or vertically [33]. This simulates slight changes in the position during ultrasound scans. The TC dataset is unbalanced; therefore, data augmentation was used to generate additional samples for the minority class (polyp images) [34]. In this study, the TC class images were doubled for each original image, and the normal class images were tripled for each original image. Thus, the training-phase dataset became the TC class 5128 and the normal class 6300.

The held-out test set was not used in developing the model. Also, the test set was not subjected to any augmentation, preprocessing, fitting steps, or hyperparameter tuning. Data augmentation operations were confined to the training phase of each cross-validation fold, and the validation and test sets were left unchanged to ensure an unbiased assessment of the model.

### 2.3. Enhancing Ultrasound Images

Improving the ultrasound images of TC is necessary to ensure an accurate diagnosis in the subsequent stages. High-quality images aid in TC detection. In this study, average and Laplacian filters were employed. Noise appears in sound wave images as grainy spots and random variations in pixel density. An average filter was used to improve image quality by removing artifacts and noise. The use of an average filter with an operating factor of 5 × 5 helps reduce artifacts and noise, thereby improving the image quality of TC ultrasound images [35]. The average filter uses a small matrix, known as a kernel, of size 5 × 5 to process the image. Each pixel in the image was processed by averaging the values of its neighboring pixels. The central pixel is replaced with the average value. After replacing all image pixels with the average of their neighbors, an improved image is obtained [36]. This is because it replaces each pixel with the average of its local neighborhood. To improve the ultrasound images of TC, applying a 5 × 5 average filter enhances the visibility of important structures while reducing noise interference, as shown in Equation (1).

(1) $$A\left(i,j\right)=\frac{1}{5x5}\sum_{s=-2}^{s=2}\sum_{t=-2}^{t=2}f\left(i+s,j+t\right)$$

In the provided framework, *f*(*i*,*j*) represents the input, *A*$\left(i,j\right)\;$ indicates the output, and *5x5* symbolizes the total pixel count.

The Laplacian filter is an image processing technique used to enhance edge details, making it particularly useful for showing boundaries and increasing contrast in ultrasound images of TC. The primary purpose of the Laplacian filter is edge detection. It identifies rapid changes in pixel intensity that correspond to image edges. Edges are abrupt transitions from dark to light or vice versa, and are crucial for delineating structures and boundaries in medical images, such as tumors in ultrasound images of the thyroid. Applying the Laplacian filter to an image computes the second derivative of pixel intensities, thereby emphasizing regions with rapid intensity changes. The negative center pixel in the filter kernel amplifies the difference between neighboring pixel values, emphasizing the edges [37]. This effect increases the contrast along the edges, making them more pronounced. The Laplacian filter effectively enhances the edges of structures in ultrasound images, such as thyroid nodules, for TC diagnosis. This enhancement makes it easier for AI techniques to identify and delineate the affected areas. The increased contrast along the edges improves visualization and aids in diagnosis, as shown in Equation (2).

(2) $$\nabla^{2}\;f(i,j)=\frac{\partial^{2}\;f}{\partial \;i_{2}}+\frac{\partial^{2}\;f}{\partial \;j_{2}}$$

They used a second-order differential equation. represented the Laplacian operator as $\nabla^{2}\;f$, with indices i and j referring to the spatial coordinates of the pixels.

Finally, the outcomes of the two image filters are combined using the 8-version of the digital Laplacian filter as follows:

(3) $$L(x,y)=(\sum_{s=-1}^{1}\sum_{t=-1}^{1}A(x+s,y+t))-9A(x,y)$$

Figure 2b shows some TC dataset ultrasound images after applying image enhancement techniques.

### 2.4. The Improvement of the DenseNet169 and VGG19 Models

The ImageNet weights were strategically used with a pretrained model. Both DenseNet169 and VGG19 early convolutional layers are ImageNet-trained and have universal visual primitives, edges, gradients, and textural discontinuities that work well in ultrasound analysis. Such a head start is critical because most medical imaging datasets are modest. Once the models are initialized, they are fine-tuned on the thyroid ultrasound dataset, and the deeper layers refine their representations to capture the sonographic morphologies of echogenicity change and microcalcification. The heads of the classifiers were removed and replaced with global average pooling, thereby making the networks feature extractors rather than end-to-end classifiers. This mixed-method initialization maintains learning transfer by providing specificity for thyroid cancer detection.

The virtues and burdens of models such as DenseNet169 and VGG19 are their architectural weights. In thyroid ultrasound, their hierarchical structure provides fine-grained resolution of tissue structure, distinguishing between indiscriminate shadowing and unmistakable calcification. However, this richness requires a calculative supremacy that is excessive, even lavish, to burden, so to speak, on the delicate, even diffuse, repertoire of a sonographic image [38]. These canonical structures could not be accepted by us; they were sculpted. In the process of sculpting them, we needed to be particularly careful to avoid deleting unnecessary computational pathways and preserve the layers that produce decisive, clinically salient features. It is a form of surgical pruning guided by the principle that efficiency should never compromise diagnostic acuity in medical imaging.

The choice of DenseNet169 and VGG19 was informed by the fact that these architectures are philosophically in nature and align with the diagnostic requirements of thyroid ultrasound interpretation. Both networks use ImageNet-pretrained weights, which, in early convolutional filters, capture universal visual primitives, including edges, gradients, and texture discontinuities, which are meaningful even when the domain is no longer a natural image but is instead sonographic data. This inheritance is important. Medical imaging data are rarely rich, and a pretrained initialization provides a stable visual vocabulary on which specialized learning can build. However, the two backbones can interpret images differently. VGG19 is structurally clear, with its convolutional hierarchy arranged in an orderly manner; therefore, it is sensitive to changes in boundary continuity and echogenicity. In contrast, denseNet169 with dense connectivity enables the reuse of multiscale features and maintains weak internal textures or microcalcification patterns. The framework achieves this balance by combining these complementary representations with the ViT encoder, providing fine-grained local morphology with global contextual argumentation and an analytical balance comparable to that of practicing radiologists.

The VGG19 structure is notoriously homogeneous, consisting of a series of convolutional and pooling layers. This simplicity renders its optimization an exercise in determining the diminishing returns. Table 2 shows that the early blocks (through Conv3) cannot be negotiated. They build basic gradient maps and edge detectors, which constitute the alphabet of any later interpretation. Nonetheless, the deeper convolutes (Conv4 and Conv5), although theoretically capable of learning high-order abstractions, seem to over-parametrize our domain. We discovered that reducing the number of convolutional filters in these later stages by 30–40% does not degrade feature performance in subsequent tasks. Its classification architecture was overlaid with global average pooling by removing the final, fully connected layers. This change shifts the network’s mission from direct classification to pure and effective feature extraction, producing a lean yet powerful 512-dimensional description [39].

DenseNet169 posed a more complex challenge. Its power lies in its dense connectivity; each layer receives input from all previous layers, enabling rich feature reuse. In this case, pruning does not refer to the elimination of successive stages as much as the scrambling of the growth inside every dense block. We retained the first convolutional layer and the block-to-block transition layers because they regulate the required compression and downsampling of the feature maps. At the scale of the dense blocks, we utilized the fact that the number of output channels per layer (the growth rate) can be reduced by 25. This balances the exponential channel expansion, which is computationally expensive [40].

Most importantly, we retained all skip connections; the integrity of this gradient highway was paramount. Global average pooling replaced the last classification layer. The outcome, as shown in Table 2 and Table 3, is a model that retains its typical multiscale feature fusion but in a more efficient and focused manner.

The VGG19 pruning targeted deep Conv4–5 blocks with many filters. Reducing the number of filters by 30–40% and replacing the classifier with a global average grouping resulted in the significant reduction you see—nearly half the parameters were removed while maintaining feature quality. DenseNet169 was more complex. Reducing the growth rate from 32 to 24, pruned each dense block without cutting critical skip links. A 25% reduction in the number of channels per layer translates to an overall parameter reduction of 31.5%, as shown.

### 2.5. Principal Component Analysis of Feature Selection

High-dimensional feature representations generated by convolutional neural networks often exhibit significant redundancy, as many channels encode interconnected visual patterns. To create a more concise and statistically stable representation, principal component analysis (PCA) was applied to the extracted convolutional feature vectors before they were used by subsequent classifiers or integrated with contextual features derived from transformers. PCA performs an orthogonal linear transformation that rotates the original feature space into a new coordinate system whose axes correspond to the directions of maximum variance [41].

Formally, given a central feature matrix $F\in R^{n\times d}$, where *n* represents the number of samples and *d* denotes the dimensionality of the extracted feature vectors.

PCA calculates the eigenvectors of the covariance matrix of the feature distribution as Equation (4):

(4) $$\Sigma =\frac{1}{n-1}F^{⊤}F$$

where $\Sigma \in R^{d\times d}$ represents the covariance matrix that captures the correlations of pairwise features. Analysis of the eigenvalues of this matrix yields a set of orthogonal eigenvectors and their corresponding eigenvalues as Equation (5):

(5) $$\Sigma v_{i}=\lambda_{i}v_{i}\;$$

where $v_{i}$ is the eigenvector *i*, and $\lambda_{i}$ its eigenvalue. The eigenvectors define the directions of the principal components, while the eigenvalues define the variance explained by each component. The principal components are then ranked in descending order according to their eigenvalues.

Instead of specifying a fixed dimension for the reduced feature space, the retained components were chosen to preserve the cumulative variance. Specifically, the smallest number of principal components k that achieve in Equation (6):

(6) $$\frac{\sum_{i=1}^{k}\lambda_{i}}{\sum_{i=1}^{d}\lambda_{i}}\geq 0.95$$

Specifically, the transformation preserves the minimum set of key axes needed to retain 95% of the cumulative variance of the original feature distribution.

This variance-preservation strategy compresses the representation while maintaining the dominant statistical structure of the extracted features. The resulting reduced feature vector is non-correlated and dimensionless, minimizing multicollinearity while preserving the information variance captured by convolutional encoders. This compressed representation is then used where dimensionality reduction is required within the framework, ensuring a consistent feature space and preserving variance for subsequent modeling stages.

### 2.6. Configuration of XGBoost and ANN Classifiers

ANN and XGBoost classifiers were constructed as optimized, non-convolutional baselines to determine the discriminative power of the extracted CNN features alone. Each was set to present a substantial and clear challenge. The search of the validation set showed that the final configuration was a binary logistic objective, a maximum tree depth of 6, a learning rate (eta) of 0.1, and 1000 estimators [42]. It was scaled using subsampling (subsample and colsample by tree at 0.8) and L2 regularization to guarantee generalizability [43]. This forms a highly powerful interaction-conscious model that considers features as tabular data.

ANN: A small, fully connected network trained to take the same feature vectors reduced using PCA [44]. The model was trained using a learning rate of 1 × 10^−4^ and binary cross-entropy loss. This design offers a rich and spatially agnostic neural benchmark [45]. These models were not selected randomly [46]. Their canonical, discipline-enforced implementations set a strict performance limit on traditional feature-classifier paradigms, thereby enabling the unambiguous quantification of the transformative impact of our ViT-based global contextual fusion [47].

### 2.7. Hybridizing ViT-E with DenseNet169 and VGG19: A Syncretic Architecture for Thyroid Nodule Parsing

This proposed a dual-pipeline hybrid architecture to meet the clinical need for the localization of morphological details and the global contextualization of anatomy. We implemented two complementary systems: the first was a combination of enhanced ViT-E with DenseNet169, and the second was a combination of ViT-E with VGG19. Both models exploit the special inductive bias of their convolutional networks to extract local features, leverage the ViT-E encoder to capture global relations, and perform the final classification. This design enables comparative analysis and provides a basis for a robust ensemble-based diagnosis [48].

To provide a comprehensive comparative analysis, we developed and evaluated two distinct hybrid architectures in parallel: ViT-E with DenseNet169 and a separate ViT-E with VGG19.

**Architecture 1: DenseNet169–ViT-E Hybrid**

The model processes an input ultrasound image tensor $X\in R^{H\times W\times 1}$.

**Stream A:** Local feature extraction using DenseNet169. A high-dimensional, multiscale feature vector is obtained via the optimized DenseNet169 backbone, which uses dense connectivity to maintain fine-grained textual patterns, as shown in Equation (7):

(7) $$f_{\mathrm{Dense}}=F_{\mathrm{Dense}}(X),f_{\mathrm{Dense}}\in R^{d_{\mathrm{dense}}}.$$

**Stream B:** ViT-E Global Context Encoding. Simultaneously, the ViT-E encoder processes the images. It is tiled into NN non-overlapping patches $N=H\times W/p^{2}$ to which positional encodings are added [49]. The ViT-E encoder (*L* of multi-head self-attention (MHSA) and feedforward networks) produces a sequence of contextualized token representations. They are summarized (e.g., through global average pooling) into a global feature vector as shown in Equation (8):

(8) $$f_{\mathrm{glob}}=\mathrm{Pool}(Z^{\left(L\right)}),f_{\mathrm{glob}}\in R^{d_{\mathrm{glob}}}.$$

Fusion and Classification. The local compressed $f_{\mathrm{Dense}}$ and the global context $f_{\mathrm{glob}}$ are combined with learned, adaptive projection as Equation (9):

(9) $$f_{\mathrm{fused}}=\phi (W_{\mathrm{Dense}}f_{\mathrm{Dense}}^{\mathrm{red}}+W_{\mathrm{glob}}f_{\mathrm{glob}}+b_{f}),$$

where *ϕ* is a ReLU activation, this combination representation is fed through the classification head of the ViT-E, a multilayer perceptron (MLP) to generate logits as Equation (10):

(10) $$o=W_{c}\cdot \mathrm{Dropout}(\mathrm{LN}(f_{\mathrm{fused}}))+b_{c}.$$

A softmax activation yields the final predictive probabilities as Equation (11):

(11) $$\left(y=c|X\right)=\frac{\exp\left(o_{c}\right)}{\sum_{j=1}^{C}\exp\left(o_{j}\right)}$$

The entire framework was developed using a shared-training methodology. The merging layer allows gradients to flow back into the convolutional neural networks, creating features that are not only unique in their own right but also complement the overall narrative provided by the transformer-based model [50]. As a result of this rigorous optimization strategy [51], the model does not simply function as a collection of independent experts but rather becomes a single, comprehensive cognitive framework that not only visualizes the thyroid nodule but also understands the full range of probabilities for its potential transformation into a malignant tumor [52].

The final probability distribution for the two categories (thyroid cancer and normal) was obtained using a softmax function.

**Architecture 2: VGG19-ViT-E Hybrid**

The second model has a similar scheme, with DenseNet169 being replaced by VGG19.

**Stream A:** Local feature extraction using VGG19. The underlying VGG19 architecture is optimized to generate a hierarchical feature representation, focusing on edge continuity and local spatial structure, as in Equation (12):

(12) $$f_{\mathrm{VGG}}=F_{\mathrm{VGG}}(X),f_{\mathrm{VGG}}\in R^{d_{\mathrm{vgg}}}.$$

**Stream B and fusion:** The global context path (ViT-E) is identical to the path in Structure 1 and shares the same encoder weights. The fusion follows the same pattern, with separate trainable projection weights. Classification is performed in the same way via the ViT-E head [53].

**Creating Spatial Detail Enhancement Block layers and headed self-attention in the enhanced ViT:**

The ViT-E route involves a radically different analysis. The image X is partitioned into N non-overlapping patches of size p×p [54].

Each patch $x_{u}$ is projected in a D-dimensional embedding linearly and flattened as Equation (13):

(13) $$z_{u}^{\left(0\right)}=E\cdot \mathrm{Flatten}\left(x_{u}\right)+b_{e},z_{u}^{\left(0\right)}\in R^{D}$$

where E is a trainable projection matrix and $b_{e}$ a bias term. Fixed sinuoidal positional encodings were added to this sequence of patch tokens $p_{u}\in R^{D}$ they are added in order to give to it spatial order as Equation (14) [55]:

(14) $$Z_{u}^{\left(pos\right)}=z_{u}^{\left(0\right)}+p_{u}$$

Once processed, the $Z_{u}^{\left(pos\right)}$ string of symbols is passed to the L-layer encoder of the ViT-E converter. The MHSA function represents the core of each L-layer encoder, as shown in Equation (15):

(15) $$Q_{h}^{l}=Z^{\left(l-1\right)}W_{Q}^{h},K_{h}^{l}=Z^{\left(l-1\right)}W_{K}^{h},V_{h}^{l}=Z^{\left(l-1\right)}W_{V}^{h}$$

The attention for head h is computed as Equation (16):

(16) $${\mathrm{Attention}}_{h}(Q_{h},K_{h},V_{h})=\mathrm{softmax}\left(\frac{Q_{h}K_{h}^{⊤}}{\sqrt{d_{k}}}\right)V_{h}$$

The outputs of all heads H are concatenated and projected. This process, followed by layer normalization (LN) and an MLP with residual connections, forms a transformer block as Equation (17):

(17) $$\begin{array}{cc} {Z^{\prime }}^{\left(l\right)} & =\mathrm{LN}\left(Z^{\left(l-1\right)}+\mathrm{MHSA}(Z^{\left(l-1\right)})\right)\\ Z^{\left(l\right)} & =\mathrm{LN}\left({Z^{\prime }}^{\left(l\right)}+\mathrm{MLP}({Z^{\prime }}^{\left(l\right)})\right) \end{array}$$

Through successive layers, this builds a representation where features are defined by their contextual associations. The output of the final encoder layer, $Z^{\left(L\right)}\in R^{N\times D}$, is flattened to form a global feature vector as Equation (18):

(18) $$f_{glob}=\mathrm{Flatten}\left(Z^{\left(L\right)}\right)\in R^{N\cdot D}$$

The final fusion step is the culmination of the architecture as we now have two compelling and complementary channel representations; locally, from the information contained in $f_{\mathrm{Dense}}$ and with $f_{\mathrm{VGG}}$ the aggregate of localized evidence, and globally from the $f_{glob}$ aggregated from across the entire input space. While assuming that the concatenation of these two complementary representations is a simple linear choice for equality, we implement a learnable projection with a dynamic gating process and a non-linear activated representation ϕ(i.e., ReLU) [56].

The trainable matrices $W_{red}$ and $W_{glob}$ allow the network to adaptively calibrate the contribution of each stream. For a diagnosis hinging on internal microcalcifications, the network can learn to weight $f_{red}$ more heavily. For assessing gross extrathyroidal invasion, the global contextual salience in $f_{glob}$ may be prioritized.

The unified representation $f_{fused}$ is then layer-normalized, regularized via dropout, and passed through the ViT-E’s classification head—an MLP—to produce logits o for the C target classes.

The ViT-E itself is already a heavy load—86 million parameters. It can be trained on VGG19 with 167 M parameters, achieving an inference time of 218 ms per batch. This is approximately four to five images per second, which is fine with offline analysis. The DenseNet169 hybrid is another that adds only approximately 10 million parameters to the ViT-E base while achieving an inference time of 167 milliseconds as shown in Table 4.

### 2.8. Inference and Visualization of Attention for Interpretability

The inference process is the most important step in developing a trained model as a diagnostic tool. The proposed ViT-E system, based on DenseNet169-VGG19, uses thyroid ultrasound images and produces two outputs: a classification result and a spatial-attenuation map. This map is essential for interpreting the model, which represents a composite inference. In this model, local feature extraction from DenseNet169 and VGG19 is combined with the overall contextual analysis of the ViT cipher, resulting in a clinically usable, visual representation. This map indicates the model’s readability by visually representing its decision-making logic and highlighting the anatomical regions that contributed most to its predictions [57].

The technical path is tight and does not reflect the model’s training architecture. The three main pathways that simultaneously process the input image consist of the DenseNet169 and VGG19 convolutional neural networks and the optimized ViT-E cipher. High-dimensional feature vectors were obtained using DenseNet169 and VGG19. These vectors were merged and reduced using PCA, eliminating redundancies and preserving the most distinctive feature axes [58]. The resulting compressed convolutional feature set was then merged with the ViT-E ciphertext’s overall contextual feature vector. This multimodal representation combines localized tissue and shape information with overall contextual knowledge, which serves as the input for the ViT-E classification. The final logarithms were transformed into subsequent probabilities for the TC and NC classes using a softmax function. These probabilities were then translated into a binary clinical diagnosis using an optimized decision threshold to balance sensitivity and specificity in the validation set, which was high owing to the risk of missing malignancy [59].

The Grad-CAM algorithm was used to interpret the reasons for any prediction. This method constructs a discriminative prominence map for each class using gradients of the target class scores from the final convolutional layer of the network. The ViT-E classification header generates a target class score yc (where c is the TC) from the features embedded in our architecture. To illustrate this, we calculated the yc gradient for the Ak feature maps of the final convolutional layers for both DenseNet169 and VGG19 models. These two networks provide spatially conserved feature maps that are essential for positioning.

For each network, the gradient-based significance weight $\alpha_{k}^{c}$ for feature map number k is calculated using global mean clustering, as in Equation (19).

(19) $$\alpha_{k}^{c}=\frac{1}{Z}\sum_{i}\sum_{j}\frac{\partial y^{c}}{\partial A_{ij}^{k}}$$

The ReLU activation function retains only the properties that positively influence thyroid cancer prediction. Custom heatmaps for both DenseNet169 and VGG19 were merged, scaled, and subjected to two-linear interpolation to produce a composite prominence map that was accurately overlaid on the original ultrasound image. This latter visualization effectively highlights the pixel regions within the convolutional paths’ field of view that are most critical to the ViT-E classifier’s decision.

The resulting attention patterns require clinical correlation and are consistent with the known ultrasound biomarkers of malignant tumors. In positive cases, attention is drawn to a narrow, focused halo around the edges of irregular or spinous nodules that visually correspond to invasive growth [60]. The nodule core exhibits a mottled or granular activation pattern, often used to indicate clusters of microcalcifications, which is a hallmark of papillary thyroid carcinoma (PTC). Notably, minor peripheral architectural abnormalities can also be highlighted, suggesting that the model can identify early extrathyroidal expansion. In contrast, the attention characteristic in normal images is diffuse. The heat map shows a smooth, low-density distribution across the homogeneous tissue, with no focal areas. In benign nodules, the focus is usually on their well-defined borders rather than their inward penetration, suggesting a benign morphological assessment [61].

Therefore, the Grad-CAM mechanism can be considered an important tool. This enables the clinician to ensure that the classification used in the system is based on anatomically and pathologically relevant image regions, thereby establishing the necessary confidence in the model. This confidence is further enhanced when the highlighted foci perfectly match the classic malignant features. By prioritizing a small, easily overlooked area that becomes significant upon examination, the model demonstrates a detectability capability that transforms the system into a collaborative diagnostic tool, which appears ambiguous when classifying. The model’s synthetic reasoning, based on a highly complex integration of pathways, is highlighted and subjected to expert scrutiny.

## 3. Results of the Proposed Techniques

### 3.1. Systems Evaluation Metrics

There are several methods for evaluating the results of AI classifiers, and the confusion matrix is widely considered an important metric. The confusion matrix is a square table that shows the number of images in the test dataset for each category. The rows and columns of the confusion matrix correspond to the actual and expected categories. The diagonal cells of the matrix represent the number of correctly classified samples, which are known as true positives (TPs). The cells below and above the main diagonal indicate incorrectly classified samples, which are further divided into false positives (FPs), false negatives (FNs) and true negative (TNeg). Equations (20)–(25) illustrate the system evaluation metrics, as follows:

(20) $$AUC=\int_{0}^{1}TPR\;\left({FPR}^{-1}\left(x\right)\right)\;dx\;$$

(21) AUC=PS^X+>S^X− 

(22) $$Accuracy=\frac{TNeg+TP}{TNeg+TP+FN+FP}*100\%$$

(23) $$Precision=\frac{TP}{TP+FP}*100\%$$

(24) $$Sensitivity=\frac{TP}{TP+FN}*100\%$$

(25) $$Specificity=\frac{TNeg}{TNeg+FP}*100$$

### 3.2. Performance Results of Pretrained CNN

This section presents an analysis of the performance results of three CNN models: DenseNet169, AlexNet, and VGG19. These models were trained using the massive ImageNet dataset, which comprises over 1,200,000 images accurately classified into more than 1000 categories. It is important to note that, while the ImageNet dataset is comprehensive, it suffers from limitations in its representation, particularly in the realm of biomedical image datasets. For example, it lacks biomedical data, such as ultrasound images from the TC dataset. The input layers of these models were designed to receive and process images from the TC dataset. Fully connected layers were used to transform these high-level features into feature vectors. Finally, the models classify each feature vector and assign it to its appropriate category, demonstrating their adaptability and usefulness for TC image classification.

**Figure 4:**
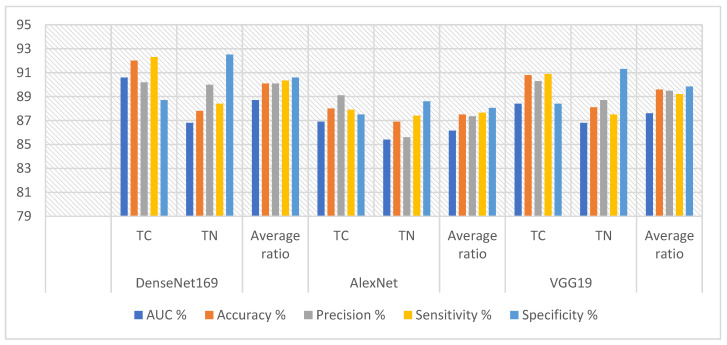
Display the results of the CNN for analysis for ultrasound to diagnose the TC dataset.

Table 5 and Figure 4 in this study provide a comprehensive overview of the performance metrics for the CNN models DenseNet169, AlexNet, and VGG19 when applied to ultrasound image analysis in a TC dataset. The DenseNet169 model achieved excellent results with an AUC of 88.7%, a sensitivity of 88.4%, a specificity of 90.6%, and an accuracy of 90.1%. The AlexNet model demonstrated competitive performance, achieving 87.35% accuracy, 88.05% specificity, 86.15% AUC, 87.65% sensitivity, and 87.5% accuracy. As for the VGG19 model, it achieved strong results with a specificity of 89.85%, an accuracy of 89.6%, an area under the receiver operating characteristic (ROC) curve (AUC) of 87.6%, and a sensitivity of 89.2%.

### 3.3. Results of the Hybrid Method Between CNN with the XGBoost and ANN Networks

The selection of the XGBoost-CNN and ANN-CNN architectures demonstrated the methodological rigor. Before proposing the shift from hybrid to transformer models, we sought to define the performance limitations of systems enhanced with CNN-ViT. Individual CNNs provided a reasonable foundation, but their accuracy was in the low 90%, which we attribute to their inherent tendency to focus on localized features at the expense of overall anatomical context.

XGBoost and ANN hybrid models were developed to test the hypothesis that more sophisticated classifiers could extract more diagnostic information from these localized features. This approach proved successful, achieving low 90% accuracy, demonstrating the potential to effectively utilize complementary convolutional features. However, despite their power, these models were subject to a localized perspective. They excelled at listing features but failed to integrate them into a unified assessment, as a radiologist would do. This is the fundamental gap that our ViT-CNN hybrid model addresses.

The model can incorporate a vision transformer encoder to capture overall contextual transformations. Distant parts of an image can influence one another, and the model predicts relationships among node boundaries, nuclei, and textures. This is not simply a replacement of the classifier but a radical shift in the applied thinking methodology by improving the model’s features for comprehensive image understanding. This section details the results of combining the XGBoost and ANN models with the CNN models for analyzing ultrasound images of thyroid cancer. Initially, CNN was used for feature extraction, which was subsequently reduced using PCA. XGBoost and ANN networks were used for classification.

**Figure 5:**
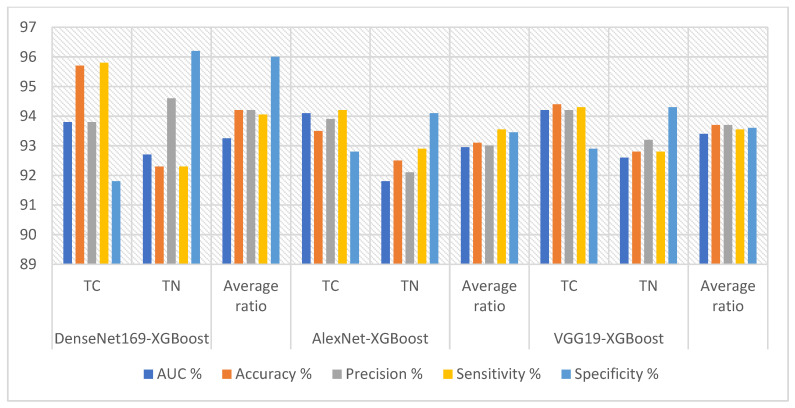
Display the results of hybrid method of CNN and XGBoost for analysis for Ultrasound to diagnose the TC dataset.

Table 6 and Figure 5 summarize the results of combining XGBoost with Dense-Net169, AlexNet, and VGG19 model features for analyzing ultrasound images of the thyroid cancer dataset. The DenseNet169-XGBoost model achieved excellent results, with an AUC of 93.25%, sensitivity of 94.05%, specificity of 96%, and accuracy of 94.2%. The AlexNet-XGBoost model achieved accuracies of 93%, 93%, 92.95%, 93.55%, and 93.1% for quality, AUC, sensitivity, and precision, respectively. The VGG19-XGBoost model performed strongly, achieving 93.6% quality, 93.7% accuracy, 93.4% AUC, and 93.55% sensitivity.

**Figure 6:**
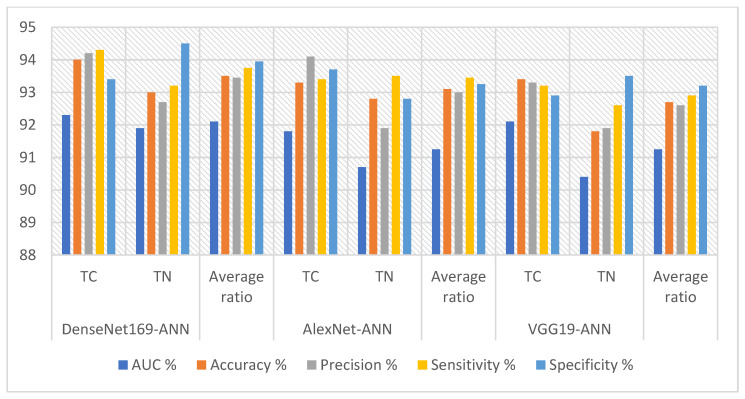
Display results of hybrid method of CNN and ANN for analysis for ultrasound to diagnose the TC dataset.

Table 7 and Figure 6 summarize the results of the ANN using features from the DenseNet169, AlexNet, and VGG19 models to analyze the ultrasound images in the TC dataset. The DenseNet169-ANN model achieved strong results, with an AUC (92.1%), sensitivity (93.75%), specificity (93.95%), and accuracy (93.5%). The AlexNet-ANN model yielded a precision (93%), specificity (93.25%), AUC (91.25%), sensitivity (93.45%), and accuracy (93.1%). The VGG19-ANN model produced robust outcomes with a precision (92.6%), accuracy (92.7%), AUC (91.25%), and sensitivity (92.9%).

### 3.4. Results of the Hybrid Method Between Fusion Features CNN with the XGBoost and ANN Networks

This section summarizes the results of the XGBoost and ANN algorithms for ultrasound image diagnosis using the TC dataset. First, the images were improved, and the required tissues were isolated to extract features. Deep feature extraction from ultrasound images using DenseNet169, AlexNet, and VGG19. The features extracted from the three CNN models were combined to form discriminators from DenseNet169-AlexNet, AlexNet-VGG19, DenseNet169-VGG19, and DenseNet169-AlexNet-VGG19 to form high-level features. PCA was used to handle the high-dimensional features. The selected features were fed to the XGBoost and ANN classifiers for classification.

**Figure 7:**
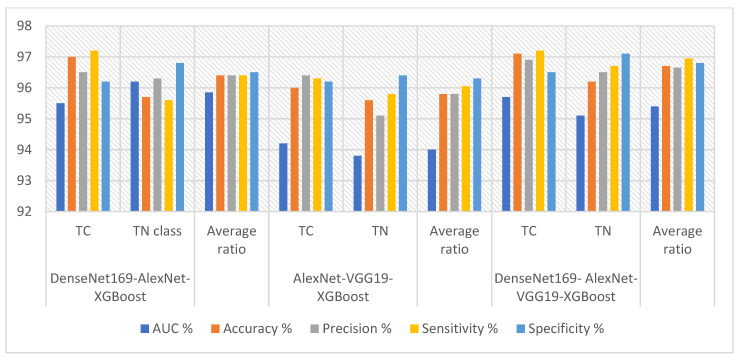
Display results of hybrid method of fusion features CNN and XGBoost for analysis for ultrasound to diagnose the TC dataset.

Table 8 and Figure 7 summarize the results of XGBoost with fusion features from the DenseNet169, AlexNet, and VGG19 models for analyzing ultrasound images in the TC dataset. The Dense-Net169-AlexNet-XGBoost model achieved reliable outcomes with specificity (96.5%), accuracy (96.4%), sensitivity (96.4%), AUC (95.85%), and precision (96.25%). The AlexNet-VGG19-XGBoost model yielded effective results, with a precision (95.8%), AUC (94%), specificity (96.3%), accuracy (95.8%), and sensitivity (96.05%). The DenseNet169-AlexNet-VGG19-XGBoost model achieved strong performance with accuracy (96.7%), sensitivity (96.65%), AUC (95.4%), specificity (96.8%), and precision (96.65%).

**Figure 8:**
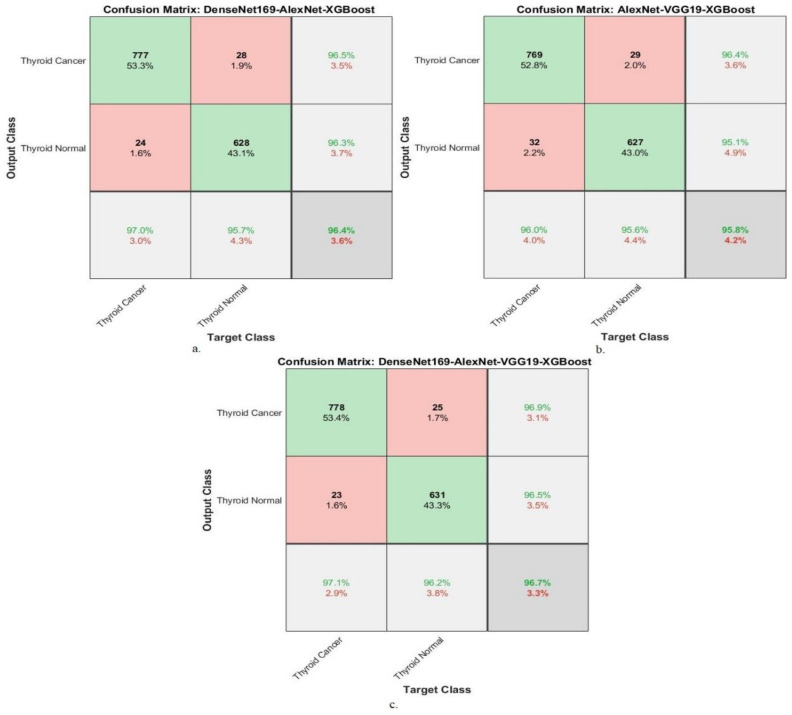
Confusion matrices for display the results of hybrid method of CNN and XGBoost for analysis for ultrasound images to diagnose the TC dataset. (**a**) DenseNet169-AlexNet-XGBoost. (**b**) AlexNet-VGG19-XGBoost. (**c**) DenseNet169-AlexNet-VGG19-XGBoost.

Figure 8 shows the confusion matrices used to evaluate the performance of the Dense-Net169-AlexNet-XGBoost, AlexNet-VGG19-XGBoost, and DenseNet169-AlexNet-VGG19-XGBoost models. Confusion matrices are valuable visual representations of system evaluation. The Dense-Net169-AlexNet-XGBoost system achieved 97% accuracy for the TC class and 95.7% for the TN class. The AlexNet-VGG19-XGBoost system demonstrated 96% accuracy for the TC class and 95.6% for the TN class. The Dense-Net169-AlexNet-VGG19-XGBoost system showed 97% accuracy for the TC class and 96.2% accuracy for the TN class.

**Figure 9:**
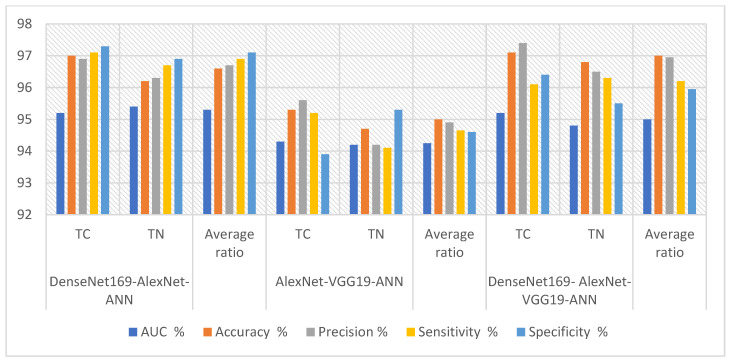
Display results of hybrid method of fusion features CNN and ANN for analysis for ultrasound to diagnose the TC dataset.

Table 9 and Figure 9 summarize the results of the ANN combined with the DenseNet169, AlexNet, and VGG19 models for analyzing ultrasound images in the TC dataset. The DenseNet169-AlexNet-ANN model demonstrated excellent performance across specificity (97.1%), sensitivity (96.9%), area under the curve (95.3%), resolution (96.6%), and fine-tuning (96.7%). The AlexNet-VGG19-ANN model achieved good results across the area under the curve (94.25%), fine-tuning (94.9%), resolution (95%), specificity (94.6%), and sensitivity (94.65%). The DenseNet169-AlexNet-VGG19-ANN model achieved strong results across accuracy (97%), fine-tuning (96.95%), area under the curve (95%), sensitivity (96.2%), and specificity (95.95%).

**Figure 10:**
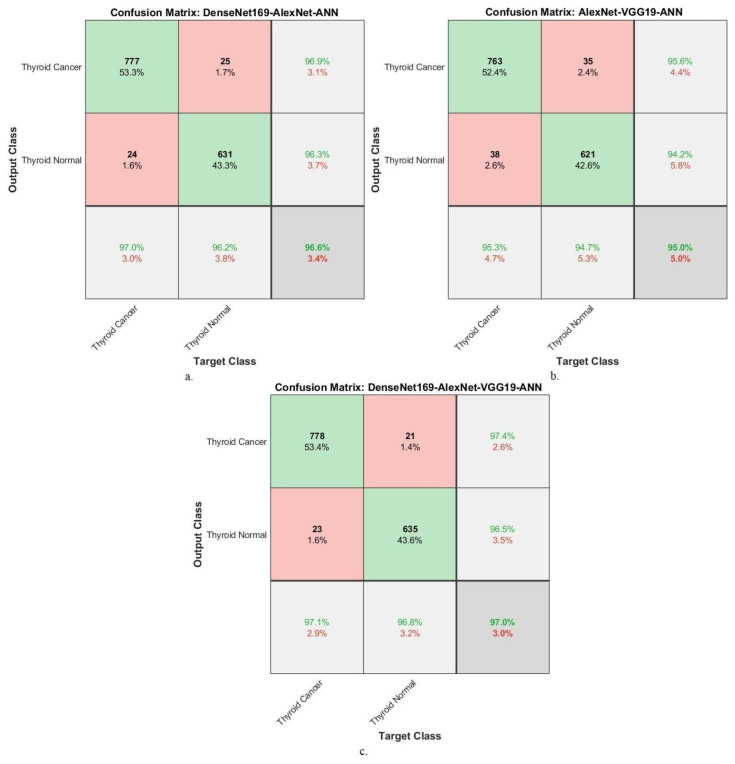
Confusion matrices for display the results of hybrid method of CNN and ANN for analysis for ultrasound images to diagnose the TC dataset. (**a**) DenseNet169-AlexNet-ANN. (**b**) AlexNet-VGG19-ANN. (**c**) DenseNet169-AlexNet-VGG19-ANN.

Figure 10 shows the confusion matrices for performance evaluation of the DenseNet169-AlexNet-ANN, AlexNet-VGG19-ANN, and DenseNet169-AlexNet-VGG19-ANNsystems. Confusion matrices serve as valuable visual representations for evaluating systems. DenseNet169-AlexNet-ANN achieves 97% accuracy for TC and 96.2% for TN. AlexNet-VGG19-ANN achieves 95.3% accuracy for TC and 94.7% for TN. The DenseNet169-AlexNet-VGG19-ANN system achieves 97.1% TC class accuracy and 96.8% TN class accuracy.

### 3.5. Syncretic Fusion and Diagnostic Precision: Evaluating the ViT-CNN Hybrids

The proposed architecture, which combines the comprehensive relational intelligence of the ViT-E network with the local morphological expertise of convolutional networks, significantly improves diagnostic performance. Previous hybrid models integrating convolutional networks with XGBoost classifiers or artificial neural networks achieved low 90% accuracy; however, our ViT-CNN models surpassed this. The results in Table 10 indicate not minor improvements but a qualitative shift in the model’s ability to decode ultrasound images of malignant thyroid tumors.

**Figure 11:**
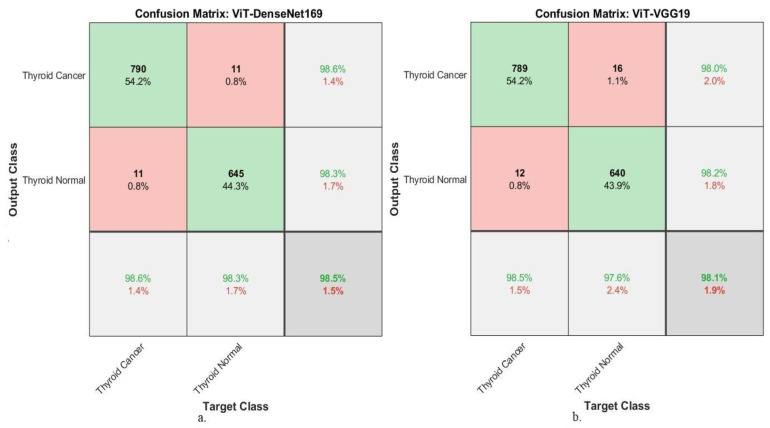
Confusion matrices for the diagnosis of thyroid tumors (**a**) ViT-DenseNet169 and (**b**) ViT-VGG19 hybrid models.

Here, we focused on ViT-DenseNet169. Its average metrics show remarkable results: 98.5% accuracy, 99.15% near-perfect specificity, and 98.9% sensitivity. This is a significant achievement in cancer detection. The system’s superior ability to identify healthy tissue in its correct location greatly reduces unnecessary patient anxiety and the need for biopsy, owing to its high specificity. However, its sensitivity ensures that malignant nodules are almost never missed. Further examination of the model’s performance across categories revealed strong performance in thyroid cancer cases, with sensitivities and accuracies of 99.3% and 98.6%, respectively. This means that when the model is used to identify a cancer case, it is correct almost every time, missing only a small percentage of genuine cancers (false negatives). The ViT-VGG19 model followed with an average accuracy of 98.1% and equally strong sensitivity (98.45%) and specificity (98.75%). The consistent superiority of the ViT-DenseNet169 model suggests that the dense feature reuse at multiple scales can be combined more effectively with the transformer’s attentional mechanisms for this task. This performance translates into tangible clinical results, as illustrated by the confusion matrices shown in Figure 11. In the case of ViT-DenseNet169, of the 801 thyroid cancer cases in the test set, only 11 were incorrectly classified as normal. This means that slightly more than 1% of malignant tumors will be missed. However, the false positives in 11 cancer cases were the normal rate for 656 healthy cases. The same trend can be observed with ViT-VGG19, with the margin of error increasing by a small margin (almost negligible) (16 false negatives and 12 false positives). Here, the clinical reality intersects with practical realities. The margin of error in these models was minimal. Not every misclassification indicates system failure. However, it indicates the most ambiguous borderline cases in the dataset—the very images that might make a human expert pause and require further investigation. The models do this not by playing it safe but by making bold and appropriate distinctions in the vast majority of cases.

Hybrid ViT-CNN models have improved the accuracy of the two most important metrics—sensitivity and specificity—to over 98.5%, a high level of expert agreement. This means that previous architectures, at least to the extent possible, were limited by their inability to provide a complete synthesis of local pixel-level evidence and a contextual understanding of the image. This is directly addressed in transformer-based data fusion by developing a cognitive model that better reflects radiologists’ integrative and interactive thinking. The result is not only a statistically superior model but also a more reliable and clinically understandable one, where its few errors are as significant as the many correct diagnoses.

### 3.6. Training–Validation Evaluation and the Role of Data Augmentation in Transformer-CNN Hybrid Diagnosis Models

A comparison between Table 11 reveals that the learning process for the hybrid models is informative. Even without additional data, the ViT-DenseNet169 model already achieves consistent diagnostic performance, with an average accuracy of 94.3% and an AUC of 93.6%. The ViTVGG19 model is presented next, with an accuracy of 93.4%. All these findings imply that transformer-CNN fusion can extract meaningful morphological and contextual information of thyroid ultrasound images despite low training variability. However, once augmentation is applied, the models’ behavior is significantly altered. ViT-DenseNet169 and ViT-VGG19 achieved performances of 98.5% and 98.1%, respectively. The answer is simple: augmentation introduces networks to realistic probe orientation, tissue presentation, and grayscale texture variations. The models do not simply learn the appearance of malignancy but also its numerous visual variations.

**Figure 12:**
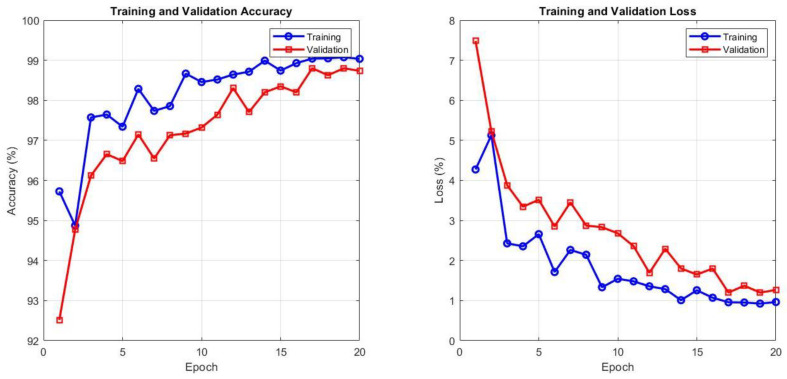
Training and validation accuracy and loss curves of the ViT-DenseNet169 hybrid model.

The ViT-DenseNet169 hybrid exhibited a very stable learning behavior, as shown in Figure 12. In the initial three epochs, the validation accuracy increased from 92.5% to 96.6%, whereas the training accuracy was deliberately increased during the first three epochs, which is a good indicator that the model is not merely memorizing. The disparity between training and validation accuracy was narrow at all steps, with a convergence gap of approximately 2%. The loss curves are most revealing: the validation loss decreases in the same direction as the training loss and then levels off at approximately epoch 10, with no indication of creeping upwards. This is a characteristic of a model that generalizes rather than parrots. At epoch 20, the architecture achieves the highest accuracy of 99.5% on training and 97.3% on validation, evidently striking a balance between the transformer’s global view and DenseNet169’s feature reuse without overfitting.

**Figure 13:**
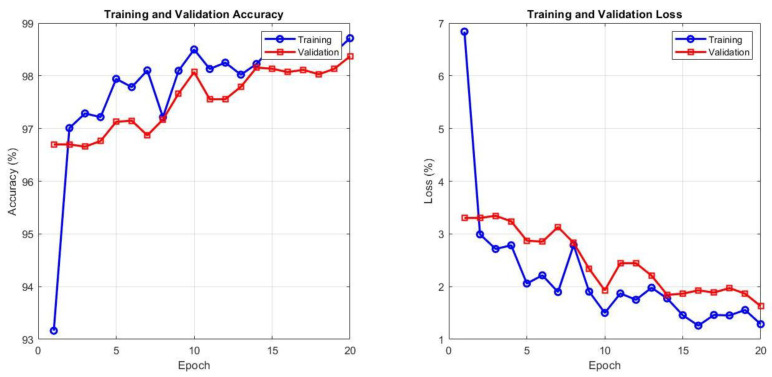
Training and validation accuracy and loss curves of the ViT-VGG19 hybrid model.

The ViT-VGG19 hybrid exhibits a slightly different pattern of fast saturation and stability, as shown in Figure 13. The training accuracy stabilized at 98% in epoch 8 and essentially stagnated afterward, whereas the validation accuracy settled at 96.7% in epoch 3 and remained unchanged. The validation loss even goes lower than the training loss after the fifth epoch and stays there, indicating that the representations of the model are transferred to the unknown data with almost perfect accuracy.

Table 12 presents the results that provide clear insights into the behavior of both hybrid architectures with respect to learning and generalization. Training of the ViT-DenseNet169 model indicates excellent stability on the training set, with an AUC of 99.17% and an accuracy of 99.14%, and very close precision, sensitivity, and specificity of 99.1%. These values indicate that the model identifies discriminative patterns in thyroid ultrasound images with high confidence. However, the validation metrics report a less tumultuous tale. The accuracy decreases to 98.74%, with an AUC of 98.85%, and the other indicators remain well balanced in the range of 98.5% to 98.9%. The fact that the training and validation curves are separated by a small margin suggests that the model has acquired generalizable representations rather than memorizing the training samples.

The same trend is observed with the ViT-VGG19 structure, but at slightly worse levels. The model achieves 98.50% and 98.43% accuracies and AUCs on the training set, with nearly identical precision, sensitivity, and specificity. The validation performances are also consistent: 98.15% accuracy, 98.10% AUC, and slight variations in the remaining indicators.

### 3.7. Mapping the Logic of Doubt: Grad-CAM as an Interpretive Lens for Thyroid Ultrasound

**Figure 14:**
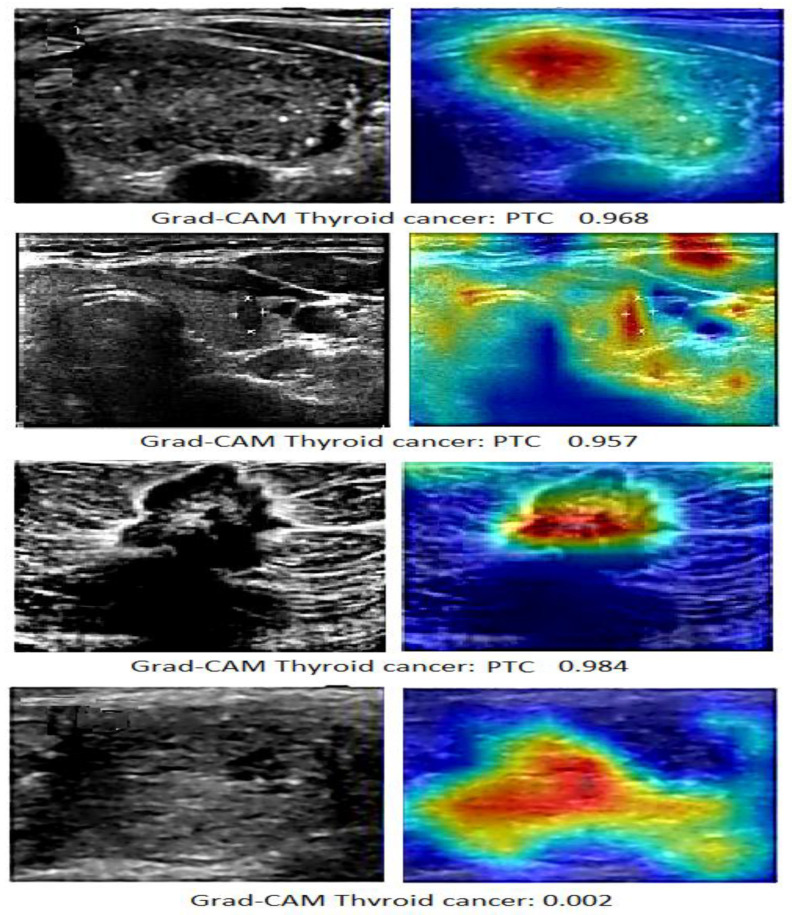
Grad-CAM representations for each ViT-DenseNet169 model, illustrating activation patterns in thyroid ultrasound images, whether the lesion is central, peripheral, infiltrative, or benign.

The interpretation of thyroid ultrasound findings lies in a narrow gray area between uncertainty and certainty. Decisions are based on a few millimeters of abnormality, slight echogenicity, and borderline frequencies. Interpretation is not merely a review but a fundamental diagnostic construct. Grad-CAM provides a summary of the deep model’s probabilistic outputs for spatial clinical inference by allowing the query of how the deep model arrives at its conclusions. The illustrative Grad-CAM visualization examples of the proposed ViT-DenseNet169 model (shown in Figure 14) support this discussion.

The first sample in Figure 14 (Papillary Thyroid Carcinoma (PTC) = 0.968) shows a well-defined hypoechoic nodule with a central dense activation area. The Grad-CAM heatmap showed a small red center precisely within the lesion border, with minimal extension into the surrounding tissue. This tendency is typical of lesion-based inference and suggests that the model identifies internal structural disturbances rather than the overall tissue. This localized activation is clinically associated with high-risk biomarkers, such as marked hypoechoicity and internal heterogeneity, typically associated with PTC. The confidence score was high and consistent with the visual evidence, confirming the tumor’s malignant diagnosis.

The second case (PTC = 0.957) shows another, but no less educationally significant pattern of activation. In this case, the Grad-CAM system identified the peripheral border of the nodule, and the second hotspot extended into the intercapsular-visceral interface. This coronal activation pattern indicates that the focus is on the irregularity of the border and the interaction within the capsular tissue, not just on the core of the lesion. This is a concerning aspect for specialists because peripheral focus is often associated with capsular invasion or early extrathyroidal extension. The spatial distribution of attentional signals suggests biologically aggressive behavior rather than simply localized malignancy, despite the high confidence score.

The most extensive and intense malignant cell activation was observed in the third case (PTC = 0.984). The Grad-CAM map extended beyond the central lesion, forming a directional activation field that distorted the expected tissue level. This field effect is not accidental; rather, it demonstrates the model’s sensitivity to asymmetric growth and potential secondary effects. These trends are consistent with infiltrative malignancies, in which disease progression extends beyond the visually dominant nodules. The near-definitive model output is explained by the broad, consistent coverage of the activation map, reflecting clinical concerns about extrathyroidal spread.

However, the fourth case (PTC = 0.002) represents only diagnostic adjustment. There was no discrete suspicious nodule on ultrasound, and the Grad-CAM map showed diffuse, low-intensity activation without any focal focus. Attention is focused on the overall tissue profile rather than on the lesions themselves. This non-obsessive orientation is diagnostically significant, indicating that the model does not raise any undue suspicion. Clinically, this behavior supports classifying the case as benign or normal and reinforces confidence in the negative results.

Figure 14 demonstrates that the ViT-DenseNet169 model is not merely an image classification model but also a spatial inference model. Different pathological conditions are associated with central nuclei, peripheral halos, gas fields, and subtle diffusion of attention. Therefore, Grad-CAM acts as a graphical representation of diagnostic ambiguity and confidence, ensuring that the algorithm’s decision is linked to known thyroid biomarkers. In this way, deep learning transforms from a purely opaque diagnostic tool into a more transparent clinical partner, capable of simultaneously alerting and reassuring at the point of care.

### 3.8. Quantifying Interpretability: Spatial Congruence Metrics for Grad-CAM in Thyroid Nodule Analysis

In addition to the qualitative assessment, quantitative measures of spatial concordance between the model focus and the expert’s clinical boundaries were provided. This transforms the interpretation from a mere visual impression to a quantifiable diagnostic examination. To establish a dual-focus mask *A* and isolate the pixels where the model’s decision-making logic was concentrated, the threshold for each malignancy was determined using the 90th percentile *H* of the Grad-CAM heatmap. This mask was then compared with the expert-provided reference manual segmentation mask *M*.

Two complementary measures were calculated for each participant: The union intersection value (IoU) was used to measure the total overlap between the model, lesion area, and actual lesion, according to Equation 26:

(26) $$\mathrm{IoU}=\frac{|A\cap M|}{|A\cup M|}$$

A value close to 1.0 indicates a high level of concordance, where the model’s focus area coincides with the expert’s anatomical boundaries. A low IoU value indicates a discrepancy that must be contextualized. This may indicate an error or, as in our case, prioritizing certain malignant features on clinical grounds, such as peripheral tissue invasion rather than nodule size. The percentage of active pixels in the nodule (PAP) is a measure of the model’s focus, as defined by Equation (27). It answers a crucial clinical question: Of the areas identified by the model as highly prominent, what percentage actually lies within the pathological zone?

The PAP in the nodule is a measure of model focus, according to Equation (15). It answers a critical clinical question: Of the areas identified by the model as highly prominent, what percentage actually lies within the pathological zone?

(27) $$\mathrm{PAP}=\frac{|A\cap M|}{|A|}\times 100\%$$

Table 13 presents the results of our proposed framework. The case focused on the node center (PTC = 0.968), achieving a high IoU (0.72) and PAP value for active pixels in the node (91), confirming that the model was anchored correctly to the node center with minimal dispersion. The peripheral case (PTC = 0.957) showed significant IoU variability (0.65), but a PAP value of 78%, supporting the 22% of the focus that was not part of the nucleus described at the capsular-visceral boundary. The infiltrating case (PTC = 0.984) showed an IoU variability of 0.58 and PAP of 62. This moderate overlap and large extralesional profile provide reproducible quantitative evidence for the model in identifying a clinically significant potential infiltration pattern.

This quantitative scrutiny goes beyond simply validating tissue prominence maps; it establishes a diagnostic dialogue between models and clinicians. The high IoU and PAP indicate the model’s confidence in localization in classic cases. When associated with aggressive tissue, a low PAP may suggest the model’s ability to identify high-risk, subtle features that require more thorough examination. Therefore, these two measures elevate the Grad-CAM visualization to the next level and connect it to the diagnostic reasoning process, providing not only an answer but also a quantitative spatial basis.

## 4. Performance of the Models in Discussion and Comparison

Diagnosing thyroid cancer using ultrasound presents a significant clinical challenge because of the often indistinct and variable appearances of malignant nodules. This complication necessitates the use of diagnostic tools that not only have high predictive power but also yield clear and interpretable clinical decisions that can be explained to clinicians.

One unresolved issue in previous studies is the lack of correlation between local feature extraction and overall image recognition methods. Most current deep learning models are either local histological classifiers or contextual segments; however, they lack an integrative mechanism for linking subtle morphological details to the overall anatomical context of the glands. This dispersion in perception limits diagnostic accuracy, particularly in early-stage or borderline cases, and negatively affects clinical accuracy by providing predictions without spatially consistent interpretations.

The existing literature review reveals that previous studies have some methodological shortcomings, as shown in Table 14. Although CNN-based models, such as VGGNet and multi-CNN clusters (Sujini et al., Zhang et al., and Vasile et al.), have proven effective for inductive classification, they inherently favor local patterns over long-range relational inference. This limitation sometimes manifests as a trade-off between sensitivity and specificity or as poor performance on small or atypical nodes (Rho et al.). Subsequent developments, such as segmentation-capable hybrid CNN-Transformer models (Li et al.) or fusion networks that combine convolutional neural networks with other classifiers, such as XGBoost or artificial neural networks (Namdeo et al. and Li W. et al.), represent notable improvements. These approaches, along with our core experiments, enhanced the performance through feature integration. However, they typically employ static delayed integration strategies, in which contextual integration occurs sequentially after feature extraction is independent. This cannot be considered a simulation of the dynamic and iterative relationship between pivotal evidence and the radiologist’s overall view of the scene. Furthermore, although interpretation techniques have been used in some studies (Aljameel et al.), they are typically employed as ex-post analyses rather than as design principles, creating a gap between model performance and clinical application.

The importance of this study lies in its attempt to fill these gaps through its original architecture, namely the explicit combination of local convolutional feature extraction and global transformer-based attention in a single, end-to-end trainable model. The ViT-E hybrid models developed, especially ViT-DenseNet169, achieved a new level of performance, as shown in Table 14. These models achieve 98.9% accuracy and 99.15% specificity, which is better than the previous systems, which had an average accuracy of 98.5% across all major measures. This success shows that the sensitivity-specificity trade-off that has been the order of the day in past studies can be overcome by incorporating local and global inference. Although pooled CNNs (Zhao et al. and Vasile et al.) have proven highly accurate, they often exhibit low or nonspecific specificity, a significant demerit for screening tools, as false positives lead to unnecessary surgical intervention. This clinical imperative is specifically met by our more specific model. This is technically achieved by continuously connecting the local morphology structure to the global context during the learning process. The multiscale reuse of DenseNet169 helps detect subtle echogenic gradients and internal abnormalities, whereas the ViT-E encoder enables long-range relational prediction across features, including boundary abnormalities and tissue behavior in the vicinity. The marginally higher score for ViT-DenseNet169 than for ViT-VGG19 indicates that dense connectivity provides a more attention-clustering-congruent feature hierarchy for this task.

In this study, quantitative interpretability is proposed as one of the main validation criteria, alongside the original performance measures.

The Grad-CAM analysis critically demonstrates the model’s clinical validity. In cases with a high predicted probability of PTC, the model’s attention maps align precisely with established malignant features. A high-confidence malignant case (PTC = 0.968) shows focused central activation, aligning with internal structural disturbance. This direct correlation between high PTC scores, specific spatial attention patterns, and known pathological biomarkers underscores that the model’s superior accuracy stems from learning clinically meaningful representations rather than spurious correlations. The further integration of Grad-CAM not only highlights the model’s significant areas; it also provides an in-depth analysis of how well the model’s attention aligns with clinical evaluation. The quantitative analysis relies on two spatial concordance indicators: IoU and PAP percentage. The proposed model has high diagnostic interest identification accuracy, owing to high IoU and PAP values (e.g., 0.72% and 91% for a central malignant node). Notably, cases with low interconnection (e.g., IoU 0.58 and PAP 62% for the infiltrative pattern) provide distinct diagnostic information, indicating that the model is focused on extranodal extensions, which are signs of invasive growth and a critical risk factor. The model interprets qualitative drawings into a quantitative concordance signal, with its high performance grounded in realistic anatomical evidence. Such spatial precision is not common in previous studies.

This study has implications far beyond incremental gains in accuracy; it addresses the role of artificial intelligence in thyroid cancer screening. Ultrasound diagnosis is an art. Radiologists tend to perceive subtle indicators of unnoticeable echogenic changes, irregular margins, and tiny clusters of calcifications that, alone, are not relevant but gain significance in their spatial context within the gland. The proposed ViT-CNN hybrids follow the same interpretive reasoning, connecting local morphological evidence via convolutional networks with global contextual reasoning via transformer attention. The models obtained have diagnostic accuracy >98%, but the true implication is reliability. High specificity minimizes unnecessary biopsies, whereas high sensitivity minimizes the risk of missed malignancies. More importantly, Grad-CAM visualizations link the algorithm’s decisions to clinically recognizable structures. In practice, this will change the system from a black-box classifier to a decision-support partner that can empower clinicians and raise doubts about ambiguous cases that should be subjected to further human investigation.

This framework should be expanded in future studies to include biopsy datasets and longitudinal follow-up to monitor potential trends.

## 5. Conclusions

This study demonstrates that the main obstacle to using artificial intelligence for thyroid ultrasound imaging lies not only in the classifier’s capabilities but also in the discrepancy between how traditional models extract evidence (localized tissue fragments) and how clinicians interpret malignancies (contextual and relational judgments). The proposed hybrid ViT-E models impose a continuous interaction between convolutional morphology and transducer-based global inference. The best model, ViT-E–DenseNet169, achieved 98.5% accuracy, 98.9% sensitivity, 99.15% specificity, and 97.35% AUC, closely followed by ViT-E–VGG19 with 98.1% accuracy and 98.75% specificity—levels exceeding the strongest previous techniques. Grad-CAM analysis clearly demonstrated the model’s clinical aspects. When the PTC value reached 0.957, the model highlighted the peripheral borders and the capsule interface, a pattern indicative of invasion. Interpretable accuracy is key: Grand/Grad-CAM heatmaps were qualitatively and quantitatively assessed using the Interrelationship Exchange (IoU) and PAP indices. Typical malignant patterns exhibited anatomically coherent attention with IoU/PAP ratios of 0.72/91% (central), 0.65/78% (peripheral), and 0.58/62% (infiltrative), while benign cases showed diffuse, low-intensity activation. These results suggest that improved performance is associated with coherent spatial inference, supporting safer clinical applications.

## Figures and Tables

**Table 1:** Distribution of thyroid histopathological images across training, validation, and testing sets.

Phase	80%		Testing 20%
Types	Training (64%)	Validation (16%)	
TC	2564	641	801
TN	2100	525	656

**Table 2:** Optimized DenseNet169 and VGG19 model structures for thyroid cancer classification.

Model	Component	Original Specification	Optimized Specification	Parameter Reduction	Rationale for Preservation
**VGG19**	Blocks Conv1–3	64–256 filters, 2–4 conv layers	Unchanged	0%	Extracts foundational edges, textures, and low-level patterns critical for margin and echogenicity assessment.
	Blocks Conv4–5	512 filters, 4 conv layers	384–448 filters, 3 conv layers	~35%	High-level abstraction capacity is partially retained while mitigating over-parameterization for ultrasound’s simpler semantic space.
	Classifier	3 Fully Connected Layers	Global Average Pooling only	~90% (of classifier)	Shifts network role to feature extractor, eliminating massive redundant linear transformations.
**DenseNet169**	Initial Conv & Pool	7 × 7 conv, 64 filters; MaxPool	Unchanged	0%	Critical first-step processing of raw pixel data into initial feature maps.
	Dense Blocks (4)	Growth rate (k) = 32	Growth rate (k) = 24	~25% per layer	Maintains multiscale feature reuse paradigm while curbing computational cost of excessive channel concatenation.
	Transition Layers	1 × 1 conv & 2 × 2 pooling	Unchanged	0%	Essential for feature map compression and downsampling; key to hierarchical structure.
	Classifier	Fully Connected Layer	Global Average Pooling only	~95% (of classifier)	Redirects model output to a dense feature vector suitable for hybrid fusion.

**Table 3:** Computational comparison of original and pruned architectures.

Model	Configuration	Parameters (M)	Inference Time (ms)
VGG19	Original	143.7	156.8
VGG19	Pruned (ours)	81.2	94.3
*Reduction*		*43.50%*	*39.90%*
DenseNet169	Original	14.3	58.4
DenseNet169	Pruned (ours)	9.8	42.7
*Reduction*		*31.50%*	*26.90%*

**Table 4:** Complexity analysis of hybrid architectures versus baseline models.

Model	Parameters (M)	FLOPs (G)	Inference Time (ms)
Pruned VGG19 (standalone)	81.2	11.4	94.3
Pruned DenseNet169 (standalone)	9.8	2.3	42.7
ViT-E (standalone)	86.4	15.8	124.6
VGG19-ViT-E Hybrid	167.6	27.2	218.3
DenseNet169-ViT-E Hybrid	96.2	18.1	167.2

**Table 5:** The results of the CNN for analysis for ultrasound to diagnose the TC dataset.

Models	Classes	AUC %	Accuracy %	Precision %	Sensitivity %	Specificity %
DenseNet169	TC	90.6	92	90.2	92.3	88.7
	TN	86.8	87.8	90	88.4	92.5
	**Average ratio**	**88.7**	**90.1**	**90.1**	**90.35**	**90.6**
AlexNet	TC	86.9	88	89.1	87.9	87.5
	TN	85.4	86.9	85.6	87.4	88.6
	**Average ratio**	**86.15**	**87.5**	**87.35**	**87.65**	**88.05**
VGG19	TC	88.4	90.8	90.3	90.9	88.4
	TN	86.8	88.1	88.7	87.5	91.3
	**Average ratio**	**87.6**	**89.6**	**89.5**	**89.2**	**89.85**

**Table 6:** The results of XGBoost with CNN features for analysis for ultrasound to diagnose the TC dataset.

Models	Classes	AUC %	Accuracy %	Precision %	Sensitivity %	Specificity %
DenseNet169-XGBoost	TC	93.8	95.7	93.8	95.8	91.8
	TN	92.7	92.3	94.6	92.3	96.2
	**Average ratio**	**93.25**	**94.2**	**94.2**	**94.05**	**96**
AlexNet-XGBoost	TC	94.1	93.5	93.9	94.2	92.8
	TN	91.8	92.5	92.1	92.9	94.1
	**Average ratio**	**92.95**	**93.1**	**93**	**93.55**	**93.45**
VGG19-XGBoost	TC	94.2	94.4	94.2	94.3	92.9
	TN	92.6	92.8	93.2	92.8	94.3
	**Average ratio**	**93.4**	**93.7**	**93.7**	**93.55**	**93.6**

**Table 7:** The results of the ANN with CNN features for analysis for ultrasound to diagnose the TC dataset.

Models	Classes	AUC %	Accuracy %	Precision %	Sensitivity %	Specificity %
DenseNet169-ANN	TC	92.3	94	94.2	94.3	93.4
	TN	91.9	93	92.7	93.2	94.5
	**Average ratio**	**92.1**	**93.5**	**93.45**	**93.75**	**93.95**
AlexNet-ANN	TC	91.8	93.3	94.1	93.4	93.7
	TN	90.7	92.8	91.9	93.5	92.8
	**Average ratio**	**91.25**	**93.1**	**93**	**93.45**	**93.25**
VGG19-ANN	TC	92.1	93.4	93.3	93.2	92.9
	TN	90.4	91.8	91.9	92.6	93.5
	**Average ratio**	**91.25**	**92.7**	**92.6**	**92.9**	**93.2**

**Table 8:** The results of hybrid method of fusion features CNN and XGBoost for analysis for ultrasound to diagnose the TC dataset.

Models	Classes	AUC %	Accuracy %	Precision %	Sensitivity %	Specificity %
DenseNet169-AlexNet-XGBoost	TC	95.5	97	96.5	97.2	96.2
	TN class	96.2	95.7	96.3	95.6	96.8
	**Average ratio**	**95.85**	**96.4**	**96.4**	**96.4**	**96.5**
AlexNet-VGG19-XGBoost	TC	94.2	96	96.4	96.3	96.2
	TN	93.8	95.6	95.1	95.8	96.4
	**Average ratio**	**94**	**95.8**	**95.8**	**96.05**	**96.3**
DenseNet169-AlexNet-VGG19-XGBoost	TC	95.7	97.1	96.9	97.2	96.5
	TN	95.1	96.2	96.5	96.7	97.1
	**Average ratio**	**95.4**	**96.7**	**96.65**	**96.95**	**96.8**

**Table 9:** The results of hybrid method of fusion features CNN and ANN for analysis for ultrasound to diagnose the TC dataset.

Models	Classes	AUC %	Accuracy %	Precision %	Sensitivity %	Specificity %
DenseNet169-AlexNet-ANN	TC	95.2	97	96.9	97.1	97.3
	TN	95.4	96.2	96.3	96.7	96.9
	**Average ratio**	**95.3**	**96.6**	**96.7**	**96.9**	**97.1**
AlexNet-VGG19-ANN	TC	94.3	95.3	95.6	95.2	93.9
	TN	94.2	94.7	94.2	94.1	95.3
	**Average ratio**	**94.25**	**95**	**94.9**	**94.65**	**94.6**
DenseNet169-AlexNet-VGG19-ANN	TC	95.2	97.1	97.4	96.1	96.4
	TN	94.8	96.8	96.5	96.3	95.5
	**Average ratio**	**95**	**97**	**96.95**	**96.2**	**95.95**

**Table 10:** Performance metrics of CNN with ViT-E hybrid models for thyroid cancer diagnosis based on data augmentation.

Models	Classes	AUC %	Accuracy %	Precision %	Sensitivity %	Specificity %
ViT-DenseNet169	TC	97.6	98.6	98.6	99.3	98.6
	TN	97.1	98.3	98.3	98.5	99.7
	**Average ratio**	**97.35**	**98.5**	**98.45**	**98.9**	**99.15**
ViT-VGG19	TC	97.2	98.5	98	99.1	98.2
	TN	96.9	97.6	98.2	97.8	99.3
	**Average ratio**	**97.05**	**98.1**	**98.1**	**98.45**	**98.75**

**Table 11:** Performance metrics of CNN with ViT-E hybrid models for thyroid cancer diagnosis without data augmentation.

Models	Classes	AUC %	Accuracy %	Precision %	Sensitivity %	Specificity %
ViT-DenseNet169	TC	93.7	94.5	95.1	95.2	94.3
	TN	93.5	94.1	93.3	94.6	95.4
	**Average ratio**	**93.6**	**94.3**	**94.2**	**94.9**	**94.85**
ViT-VGG19	TC	92.7	93.5	94.5	94.2	93.1
	TN	92.5	93.3	92.2	93.4	94.5
	**Average ratio**	**92.6**	**93.4**	**93.35**	**93.8**	**93.8**

**Table 12:** Comparative training and validation performance of ViT-DenseNet169 and ViT-VGG19 hybrid models for ultrasound-based thyroid cancer diagnosis.

Dataset	Model	AUC %	Accuracy %	Precision %	Sensitivity %	Specificity %
Training	ViT-DenseNet169	99.17	99.14	99.13	99.14	99.1
	ViT-VGG19	98.43	98.5	98.45	98.44	98.47
Validation	ViT-DenseNet169	98.85	98.74	98.9	98.55	98.78
	ViT-VGG19	98.1	98.15	98.2	98.25	98.16

**Table 13:** Quantitative spatial congruence analysis of model attention for representative cases.

Case (PTC Score)	Qualitative Pattern	IoU (90% ile)	PAP (Within Nodule)	Diagnostic Interpretation
**First case** **(0.968)**	Central, focused	0.72	91%	High-fidelity localization; attention anchored to nodule core.
**Second case** **(0.957)**	Peripheral, rim-like	0.65	78%	Strong overlap with focused margin activation, suggesting capsular assessment.
**Third case** **(0.984)**	Infiltrative, extended field	0.58	62%	Moderate overlap with significant extra-nodular attention, indicating potential invasive growth.

**Table 14:** Comparison of the proposed systems with previous works.

Authors	Systems	AUC %	Accuracy %	Precision %	Sensitivity %	Specificity %
Sujini et al. [16]	six-layer CNN and VGGNet-16	94.7	-	-	-	-
Zhang et al. [18]	Multi-CNN	91	-	94	90	-
Naglah et al. [20]	CNN with Texture Patterns	87	-	-	-	97
Li et al. [21]	Eff-Unet + CNN-Fusion	85.5	86	-	-	-
Zhao et al. [22]	CNN Ensemble	94.7	-	-	-	-
El-Hossiny et al. [23]	CNN for Carcinoma Classification	94.69	-	-	-	-
Aljameel et al. [24]	EANN model	86	82	-	-	-
Wu et al. [25]	Three CNNs	82.9	-	97	77.9	-
Zhang et al. [26]	InceptionResNetV2	97.1	-	-	90	-
Wang et al. [27]	CNN with Clinicopathological Factors	78	-	-	-	-
Rho et al. [28]	CNN for Small Nodules	66	83.2	-	89.8	38.3
Vasile et al. [29]	Ensemble CNN Models	-	97.35	95.75	-	-
Our Proposed Systems	ViTE-DenseNet169	**97.35**	**98.5**	**98.45**	**98.9**	**99.15**
	ViTE-VGG19	**97.05**	**98.1**	**98.1**	**98.45**	**98.75**

## Data Availability

The datasets used to analyze ultrasound images for early detection of thyroid cancer were obtained from publicly available online repositories and can be accessed via the following link: https://www.kaggle.com/code/garesothmen/thyroid-classification/notebook (accessed on 25 May 2025).
